# Dynamic Measurements Using FDM 3D-Printed Embedded Strain Sensors

**DOI:** 10.3390/s19122661

**Published:** 2019-06-12

**Authors:** Marco Maurizi, Janko Slavič, Filippo Cianetti, Marko Jerman, Joško Valentinčič, Andrej Lebar, Miha Boltežar

**Affiliations:** 1 Department of Engineering, University of Perugia, Goffredo Duranti 93, 06125 Perugia, Italy; marcomaurizi06@gmail.com (M.M.); filippo.cianetti@unipg.it (F.C.); 2 Faculty of Mechanical Engineering, University of Ljubljana, Aškerčeva 6, 1000 Ljubljana, Slovenia; marko.Jerman@fs.uni-lj.si (M.J.); jv@fs.uni-lj.si (J.V.); andrej.lebar@fs.uni-lj.si (A.L.); miha.boltezar@fs.uni-lj.si (M.B.); 3 Faculty of Health Sciences, University of Ljubljana, Poljanska c. 26a, 1000 Ljubljana, Slovenia

**Keywords:** 3D-printing, strain sensors, embedded sensors, dynamic measurements, fused deposition modeling, smart structures

## Abstract

3D-printing technology is opening up new possibilities for the co-printing of sensory elements. While quasi-static research has shown promise, the dynamic performance has yet to be researched. This study researched smart 3D structures with embedded and printed sensory elements. The embedded strain sensor was based on the conductive PLA (Polylactic Acid) material. The research was focused on dynamic measurements of the strain and considered the theoretical background of the piezoresistivity of conductive PLA materials, the temperature effects, the nonlinearities, the dynamic range, the electromagnetic sensitivity and the frequency range. A quasi-static calibration used in the dynamic measurements was proposed. It was shown that the temperature effects were negligible, the sensory element was linear as long as the structure had a linear response, the dynamic range started at ∼ 30 μϵ and broadband performance was in the range of few kHz (depending on the size of the printed sensor). The promising results support future applications of smart 3D-printed systems with embedded sensory elements being used for dynamic measurements in areas where currently piezo-crystal-based sensors are used.

## 1. Introduction

3D-printed smart structures and intelligent systems have recently attracted significant research interest [1,2,3]. Smart structures are based on their ability to change themselves under environmental and inherent variations [4]; therefore, a dynamic nature is intrinsic [5]. Consequently, sensors and actuators are necessary to monitor and vary the system properties. In particular, dynamic strain measurements are crucial in 3D-printed aerospace components [6], medical diagnostics [7] and smart structures due to the need to monitor their fatigue life [8,9]. Additionally, the rapid growth of the 3D-printing processes, in particular their ability to print novel functional materials [6,10,11,12], such as electrically conductive printable polymers, has made it possible to realize objects with sensory characteristics [13,14]. These potentialities have been used to manufacture 3D-printed sensors, which are basically tested only for static or low-frequency measurements in the case of strain sensors [15], substituting the classic, commercial, off-the-shelf sensory elements, especially for the rapid prototyping of 3D structural electronics [16,17], and reducing the time to market and the overall development cycle [18]. Many fields of science and engineering have been involved in the development of 3D-printed sensors, measuring the angular changes in finger kinematics [19], pH and conductivity in water-distribution systems [20], sound by 3D-printed bionic ears [21] and strain by wearable sensors for home healthcare [22].

Strain measurements are essential for monitoring mechanical systems, from both the static and the dynamic points of view [23,24,25]. 3D-printed static strain sensors have been implemented using different conductive materials, including single/multi-walled carbon nanotubes (S/M-WCNTs) [26,27], graphite [28], graphene films [29] and carbon black (CB) [30] as fillers in conventional polymers. Furthermore, different technologies have been adopted to integrate sensors (including strain sensors) into 3D-printed structures [31], such as hybrid approaches [18,32,33], where, e.g., the sensor is inserted while printingl conductor infusion [2,34] and multi-material printing [35]. The capability of the latter method to simultaneously 3D print the functional (e.g., strain sensor) and the structural part has recently become an object of significant research interest [35,36]. In particular, fused deposition modeling (FDM) techniques have been rapidly improved in recent years, making it possible to easily co-print functional and structural materials at low cost [36,37,38,39].

Printing embedded strain sensors using the FDM process takes advantage of the technology itself, enabling a sensor of arbitrary shape and printable connections, while avoiding the installation/assembly and the associated problems [31]. Obviously, the FDM printing of embedded sensors has limitations, such as material inconsistencies and poor extrusion [40]; materials used in FDM printing can additionally suffer from creep, non-linearity and hysteresis [31]. However, its current and future potentials are greater than its drawbacks. Geometry (the number of gauge-end loops, strand width and gauge thickness) and build-orientation effects with respect to the static properties (linearity, hysteresis and repeatability) of FDM 3D-printed conductive graphene-based PLA strain gauges embedded in structures, using tensile-test machines, have been systematically studied (e.g., see [35]). Additionally, the conductivity and piezoresistive response (under cyclic loads) of FDM 3D-printed strain sensors have been researched, investigating the effect of the FDM print parameters (nozzle, bed temperature and layer height), using thermoplastic polyurethane/multi-walled carbon nanotube composites [41]. Piezoresistive static and cyclic (low-frequency) measurements using ink-jet and screen-printed embedded strain sensors have been performed in earlier research, adopting a bending experimental set-up [28,42]; besides, cyclic tests on wearable 3D-printed strain sensors have been carried out [22]. Bending (manually tested) low-frequency measurements have also been carried out for FDM 3D-printed strain sensors integrated into structures [35,39].

In previous research, dynamic measurements (except for low-frequency cyclic measurements, e.g., [27,29,41,43]) have not been considered. This study researched the dynamic strain measurements that are typically employed in structural dynamics using FDM 3D-printed piezoresistive sensors embedded in structures. The paper is organized as follows. In Section 2, the design and production of the test-samples are described, together with dynamic considerations about the propagation of stress waves. In addition, the theoretical explanations and assumptions of the dynamic approach used and the experimental procedure are described. In Section 3.1, the quasi-static calibration and considerations about the sensors’ response under harmonic excitations are reported. In Section 3.2, the dynamic measurements analyzed in the frequency domain are shown and discussed. Finally, the conclusions (Section 4) are drawn.

## 2. Materials and Methods

### 2.1. Materials, Specimen Design and Production

**Figure 1:**
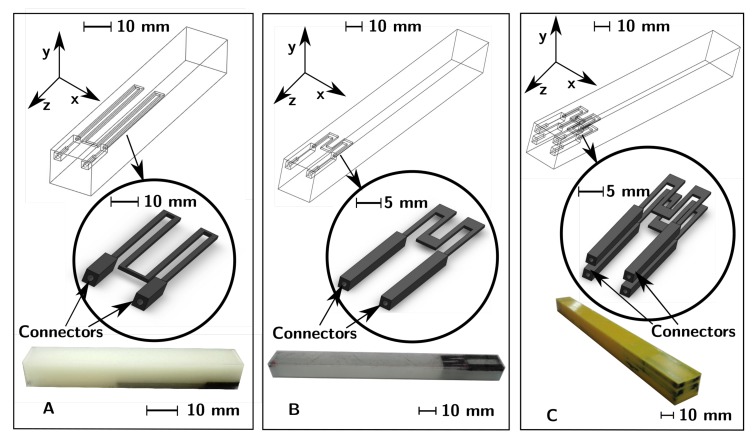
(**A**–**C**) The geometry of Samples A–C: CAD model of the samples and sensors and the 3D-printed manufactured specimens. The non-sensing material is PLA for Samples A–C, even if the colors are different.

To investigate the dynamic behavior of FDM 3D-printed strain sensors embedded in structures (3D co-printed) and the potential of a quasi-static calibration based on a dynamic approach, three different types of beam samples were manufactured by material extrusion, using a FDM printer Ultimaker 3 dual extruder (Ultimaker, Cambridge, MA, USA), choosing the Polylactic Acid (PLA) filament (3D-FilaPrint premium, diameter 2.85 mm) as the non-sensing material and the electrically conductive PLA ProtoPasta filament (ProtoPlant, WA, USA) as the functional material, which is a compound of Natureworks 4043D PLA, a dispersant and conductive carbon black (CB). The non-functional material and the sensory elements were 3D-printed in a single-step additive manufacturing process (material extrusion [44]) and in the same build cycle. For each sample, a layer height of 0.1 mm and a bed temperature of 60 ${}^{\circ }$C were used for both materials, whereas the nozzle temperatures adopted were 220 ${}^{\circ }$C and 225 ${}^{\circ }$C for the PLA and the conductive PLA, respectively. A nozzle diameter of 0.4 mm was used. The printing temperatures were selected to have a good print quality, while the layer height to maintain an acceptable layer quality and the possibility to print details of that dimension in height. Samples and sensors were printed using 100% of infill density. The direction of the printing was along the *y* axis (Figure 1), avoiding problems related to the build orientation [35]. Additionally, the direction of the printing influences the sensory elements’ behavior because of the different resistivity (see principle of measurement in the Section 2.2) along and perpendicular to the layers, as supplied roughly by the manufacturer for the conductive PLA (30 and 115 Ωcm in and out of layer, respectively); therefore, with stable printing parameters, comparable samples were 3D-printed, in which the only parameter to be identified was the sensors’ sensitivity (Section 2.2). The test specimens consisted of a PLA beam with a rectangular cross-section (12 mm × 10 mm), in which one or more conductive PLA strain sensors were 3D co-printed, i.e., the sensors were printed in the same build cycle of the structures by the dual extrusion system. The sensory elements in every sample were designed to sense the longitudinal component of strain ${\bar{\epsilon }}_{zz}$ averaged in their occupied volume, printed slightly below the surface in order to demonstrate their ability to perform dynamic measurements, even in the case of complete embedding.

The specimens (Samples A–C), with the integrated sensors, are shown in Figure 1; the strain sensors were built with connectors, represented by two cavities of length 5 mm for Sample A and 7 mm for Samples B and C. A cantilever-beam experimental set-up (explained in the next sections) was chosen to obtain a zero strain connectors zone. Additionally, silver paint between the sample’s connectors and the lead wires was used to reduce the contact resistance. The sensor element in the form of an M-shape was chosen to demonstrate the feasibility of the proposed approach. The M-shape of the sensors was designed to make the sensory elements mainly sensitive to the longitudinal component of strain ($\epsilon_{zz}$), due basically to the wire-shape of the sensors’ active part. The M-shape was preferred, instead of realizing U-shaped sensors, to obtain a strain measurement averaged in a small zone at a certain distance from the neutral plane, imitating the classical strain gauges’ behavior. Every sensor element had a width of 0.8 mm (in the plane *x*-*z*) and a thickness of 0.5 mm (*y*-direction), corresponding to five layers. The thickness of the sensors was determined considering empirically the minimum number of layers so that material discontinuity problems did not occur, in order to obtain the minimum average along the distance from the neutral plane. The sensors in Samples B and C had transversal parts with a width of 2.80 mm to reduce the cross sensitivity (*x*-direction). Sample A had a total length of 70 mm with 10 mm of clamping length. It also had one integrated sensor, whose active length was 30 mm, at 3.50 mm from the beam’s neutral plane (zero longitudinal component of stress). The total length of Samples B and C was 140 mm, with 20 mm of clamping length. In Samples B and C, a sensor, with an active length of 10 mm, was 3D co-printed at 3.50 mm from the neutral plane. Sample C also had an adjunctive sensor, equal to the other, but in the theoretical neutral plane in order to investigate the possibility to compensate the noise due to the EMI (Electro-Magnetic Interference). A greater sample length (compared to Sample A) for Samples B and C was adopted to obtain a higher strain ${\bar{\epsilon }}_{zz}$; at the same time, the sensor length was reduced.

The process parameters were tuned until the process was stable and no defects, such as material inconsistencies and poor extrusion, were found in the FDM printed samples; hence, a fixed set of printing parameters was chosen. Besides, the sensory function of the conductive element did not result to be significantly influenced by a reasonable deviation of the printing parameters, guaranteeing a good printing quality.

#### Stress-Waves Propagation and Sensor Length

The strain gauge’s length should be 1/10 (or less) of the minimum stress wavelength (maximum frequency), expected to be measured [45]. The assumption is based on the intuitive consideration that if the sensor length was comparable to the stress wavelength, the sensor output would not be accurate (it would spatially average the strain). Considering the Rayleigh beam theory [46], the maximum measurable frequencies were determined as c/λ, where *c* is the phase velocity (c=1631.3 m/s) and λ is the stress wavelength, assumed to be ten times the sensor length. For a sensor of length 30 mm in Sample A, the maximum theoretically measurable frequency is 5.4 kHz, while, for the sensors in Samples B and C, it is 16.3 kHz. The longitudinal elastic modulus *E* (Young’s modulus) and the density ρ for the PLA were experimentally found to be E=3300 MPa and ρ=1240 kg/m${}^{3}$(see Section 2.4) resulting in $c=\sqrt{E\;/\;\rho }=1631.3$ m/s.

### 2.2. Dynamic Measurements and Assumptions

The FDM 3D-printed embedded strain sensors tested in this study were based on the piezoresistive principle, i.e., the capability of an electrically conductive material to change its resistance if a mechanical deformation occurs. The resistance *R* of a conductor of uniform cross-section *A*, length *l* and resistivity ρ, neglecting the temperature effects, is given by [47]:

(1) $$R=\rho \frac{l}{A}$$

During deformation *l*, *A* and ρ could change: if only the first two vary, a behavior similar to the metallic foil strain gauges occurs; otherwise, if the resistivity change is predominant, a semiconducting material-like functioning occurs [47]. Considering the resistivity as ρ=k/(N/V), where *k* is a proportional factor, N/V is the number (*N*) of mobile electrons per unit volume (V=Al) and substituting it into the Equation (1), the resistance of the wire is expressed as [47]:

(2) $$R=k\;\frac{l^{2}}{N}$$

Computing the total differential of Equation (2) with respect to the variables *l* and *N* around the undeformed shape ($dR=\left(\partial R\;/\;\partial l\right)\;dl+\left(\partial R\;/\;\partial N\right)\;dN$) and dividing by $R_{0}$, the relation $dR\;/\;R_{0}=2\;dl\;/\;l_{0}-dN\;/\;N_{0}$ is obtained, where the subscript 0 indicates the reference condition in the undeformed shape at the initial instant of time $t_{0}$. Assuming that $dN\;/\;N_{0}=\Pi \;dl\;/\;l_{0}$, defining the gauge factor as GF=2−Π (if Π=0 metal strain gauges behavior occurs) and including the temperature-change effects, it is possible to obtain [47]:

(3) $$\frac{dR}{R_{0}}=GF\left(\frac{dl}{l_{0}}+\left(\alpha -\beta \right)dT\right)+\gamma \;dT$$

where a uniaxial stress field is considered, α and β are the coefficients of thermal expansion of the specimen and sensors, respectively; γ is the temperature coefficient of resistance; and *T* is the temperature. Assuming that the sensor’s piezoresistive behavior is linear, i.e., *GF* is not strain dependent, and that γ, α and β do not depend on the temperature or the strain, and integrating Equation (3) from the initial time (subscript 0) to the time *t*:

(4) $$\frac{R\left(t\right)-R_{0}}{R_{0}}=GF\left(\frac{l\left(t\right)-l_{0}}{l_{0}}+\left(\alpha -\beta \right)\left(T\left(t\right)-T_{0}\right)\right)+\gamma \left(T\left(t\right)-T_{0}\right)$$

Additionally, the GF was assumed to be temperature independent under ambient conditions [28]. Considering, furthermore, small displacements and a linear mechanical system, the term $\left(l\left(t\right)-l_{0}\right)\;/l_{0}$ in Equation (4) can be used to identify the strain component ${\bar{\epsilon }}_{zz}\left(t\right)$ [48]. Due to the linear assumption, the dynamic behavior can be researched for a particular harmonic load at the frequency $f_{exc}$. Assuming a harmonic change in the resistance $R\left(t\right)=\bar{R}\left(t\right)+R_{A}\;\cos\left(2\;\pi \;f_{exc}\;t+\Phi_{R}\right)$ and also the strain ${\bar{\epsilon }}_{zz}\left(t\right)={\bar{\epsilon }}_{zz,A}\;\cos\left(2\;\pi \;f_{exc}\;t+\Phi_{\epsilon }\right)$, from Equation (4), it follows that:

(5) $$\bar{R}\left(t\right)+R_{A}\;\cos\left(2\;\pi \;f_{exc}\;t+\Phi_{R}\right)=R_{0}\;GF\;{\bar{\epsilon }}_{zz,A}\;\cos\left(2\;\pi \;f_{exc}\;t+\Phi_{\epsilon }\right)+R_{0}\;\left(GF\;\left(\alpha -\beta \right)+\gamma \right)\;\left(T\left(t\right)-T_{0}\right)+R_{0}$$

As Equation (5) should be valid for any frequency ($f_{exc}$), it can be decomposed into:

(6) $$\begin{array}{ccc} R_{A}\;\cos\left(2\;\pi \;f_{exc}\;t+\Phi_{R}\right) & = & R_{0}\;GF\;{\bar{\epsilon }}_{zz,A}\;\cos\left(2\;\pi \;f_{exc}\;t+\Phi_{\epsilon }\right) \end{array}$$

(7) $$\begin{array}{ccc} \bar{R}\left(t\right) & = & R_{0}\;\left(GF\;\left(\alpha -\beta \right)+\gamma \right)\;\left(T\left(t\right)-T_{0}\right)+R_{0} \end{array}$$

It is reasonable to assume $\Phi_{R}=\Phi_{\epsilon }$, which results from Equation (6) in:

(8) $$GF=\frac{\frac{R_{A}}{R_{0}}}{{\bar{\epsilon }}_{zz,A}}$$

From Equation (7), it is evident that the mean value of the resistance is only influenced by the temperature variations. From Equation (4), the zero order of the sensor is clear and Equation (8) can be written in the frequency domain as:

(9) $${\bar{\epsilon }}_{zz,A}\left(f\right)=\frac{1}{GF}\;\frac{R_{A}\left(f\right)}{R_{0}}$$

### 2.3. Experimental Procedure

**Figure 2:**
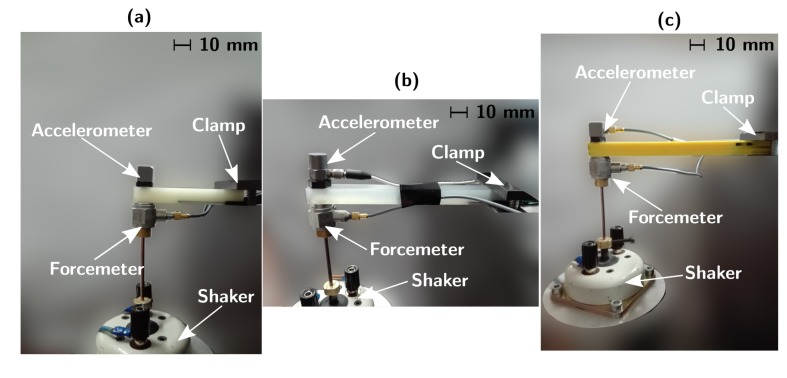
Cantilever-beam experimental set-up for dynamic measurements: (**a**) Sample A; (**b**) Sample B; and (**c**) Sample C.

Samples A–C were tested in a cantilever-beam configuration, as shown in Figure 2.

**Figure 3:**
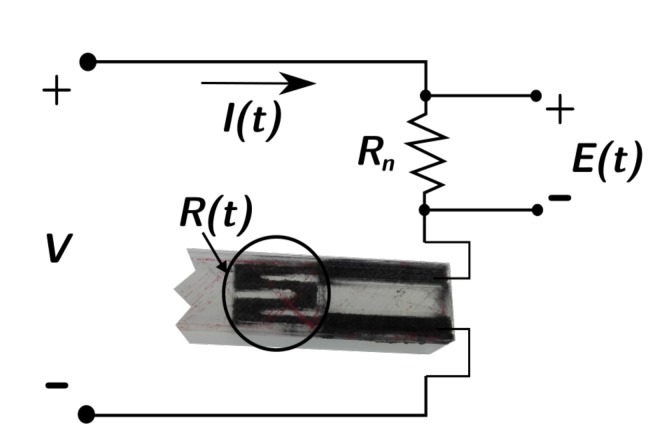
Circuit scheme of the voltage divider used to measure the resistance change in the integrated sensors.

The specimens were excited with an electrodynamic shaker (LDS V101/2), amplifying the generated voltage signal with a power amplifier (LDS PA25E). A uniaxial accelerometer (PCB Piezotronics T333B30) was installed (using wax) on the free edge surface of the beam sample (Figure 2), used to acquire the acceleration signal, seen as the mechanical output of the system. Another uniaxial accelerometer (PCB Piezotronics 352C33) was used to measure the system responses of Samples B and C. A forcemeter (PCB Piezotronics 208C01) was set up (glued) to measure the force as the input to the system and a stinger was used to regulate the height of the shaker and avoid pre-stressed conditions on the sample (Figure 2). To measure the 3D-printed sensor response, a simple voltage divider was employed [39,41], as shown in Figure 3, adopting a known commercial resistance $R_{n}$ of 10 kΩ and a supply voltage *V* of 12 V. Measuring the voltage drop *E(t)* on the known resistance ($E\left(t\right)=R_{n}\;I\left(t\right)$), the *R*(*t*) signal from the sensor is:

(10) $$R\left(t\right)=\left(\frac{V}{E(t)}-1\right)R_{n}$$

Measuring E(t) results in the identification of R(t) and (if the gauge factor (GF) is known) via Equation (8) in the identification of strain ${\bar{\epsilon }}_{zz,A}$. Since the absolute resistance changes of the 3D-printed sensors are much greater than the classic strain gauges (Ω instead of μΩ), the voltage divider was chosen instead of other circuits, such as the Wheatstone Bridge.

#### 2.3.1. Quasi-Static Tests

To determine the gauge factor (Equation (8)), the quasi-static test, which consists of exciting the structure (samples) with a harmonic load at a frequency significantly below the first natural frequency, where the structural dynamics does not influence the measurements, was prepared. The first natural frequencies of the clamped Samples A–C were experimentally identified as 217 Hz, 104 Hz, and 103 Hz, respectively. Samples A–C were excited by generating a harmonic input at 30 Hz (NI 9263 and LDS PA25E Amplifier) and controlling the force amplitude $F_{A}$ measured from the forcemeter with a PID controller implemented in Labview. To generate an integer number of cycles and to have good stability of the PID controlling system, the number of acquired points was 28,446 at the sampling frequency of 17,067 Hz. The nonlinearities in the measured R(t) were analyzed in the time and frequency domains. Sample C was additionally tested, investigating the possibility to compensate the noise (due to EMI) by the sensor in the neutral plane: measuring its resistance $R_{NP}\left(t\right)$ (Equation (10)) and subtracting $R_{NP,A}\left(t\right)$ from R(t) of the active sensor.

To perform the quasi-static calibration, the amplitude $R_{A}$ and the mean value $\bar{R}$ of the signal R(t) were acquired 100 times every 1.6667 s. With this approach, several sets of measurements, from 2 N to 4.8 N ($F_{A}$) with a step of 0.4 N, were made. The calibration curves were obtained for the samples, estimating ${\bar{\epsilon }}_{zz,A}$ by using the Euler–Bernoulli beam theory (for Samples A–C) and the amplitude $|H_{\epsilon F}\left(f\right)|$ FRF (Frequency Response Function) (for Samples B and C) obtained using the numerical model (see Section 3); the $H_{yx}\left(f\right)$ indicates the estimator of the theoretical (FRF) Y(f)/X(f), where Y(f) and X(f) are the Fourier transforms of the output and input, respectively ([49]). The GFs were computed from Equation (8) and presented in Section 3.1.

#### 2.3.2. Dynamic Tests

Once the GF was identified, we could proceed with the dynamic tests in the broad frequency band. The dynamic tests on Samples A–C were performed using the set-up shown in Figure 2. An analog signal generator (Rigol) was used to produce sine sweeps in the range 100 Hz to 500 Hz for Sample A and 5 Hz to 200 Hz and 5 Hz to 4 kHz for Samples B and C, with a logarithmic frequency increase. To acquire data from the accelerometer, the forcemeter and the 3D-printed strain sensor, a DAQ (NI 9234) connected to a Personal Computer and a Labview VI program were used, with a sampling frequency $F_{s}$ of 25.6 kHz and the time of acquisition $T_{s}=1$ s. Acquiring and post-processing the acceleration *A(t)* and force *F(t)* in the *y*-direction (coordinate system in Figure 1), the FRF $H_{AF}\left(f\right)$ was obtained. Analogously, the piezoresistive FRF $H_{R_{A}F}\left(f\right)$ was estimated, measuring the F(t), the sensor response R(t) and the value $R_{0}$ for the reference conditions (undeformed shape and $T=T_{0}=T\left(t_{0}\right)$), resulting in a force-to-strain FRF $H_{\epsilon F}\left(f\right)$:

(11) $$H_{\epsilon F}\left(f\right)=\frac{1}{GF}\;H_{R_{A}F}\left(f\right)=\frac{1}{GF}\frac{S\left\{F\left(t\right),\frac{R(t)}{R_{0}}\right\}}{S\left\{F(t)\right\}}$$

where $S\left\{\cdot \right\}$ and $S\left\{\cdot ,\cdot \right\}$ are the estimation of the Power Spectral Density (PSD) and the Cross Power Spectral Density (CPSD), respectively. The coherence function was estimated to check the system’s linearity.

The experimental FRFs were determined at three different increasing levels of input (RMS of force) to research the non-linearities, separating the global system (mechanical and piezoresistive points of view) non-linearities from those of the mechanical system itself.

The dynamic measurements were performed following the principles typically used in structural dynamics (as [45]) for determining the frequency response functions of a mechanical system.

### 2.4. Finite-Element Modeling Dynamic Simulation

**Figure 4:**
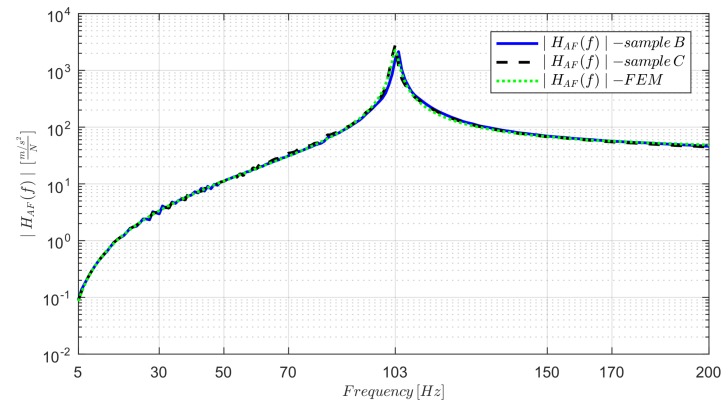
Numerical model validation in the range 5 Hz to 200 Hz for Samples B and C.

The structural FEM model of Sample B in the experimental configuration was developed, including the sensors, using the geometry shown in Figure 1. The FEM model was built to estimate the strain FRF $H_{\epsilon F}\left(f\right)$ for Samples B and C (negligible differences, as shown below), used for the calibration (as shown in Section 2.3.1) and for the sensor’s dynamic validation comparing the FRF $|H_{\epsilon F}\left(f\right)|$ amplitude to the experimental one (as shown in Section 2.3.2). The latter was estimated by using the *GFs* determined by the numerical model itself. The 3D-printed sensors’ capability to measure the strain in the frequency domain and identify the system’s natural frequencies were therefore researched. Matching the numerical $|H_{AF}\left(f\right)|$ to the experimental one, as shown in Figure 4 for the range 5 Hz to 200 Hz, the validation was also assumed for $|H_{\epsilon F}\left(f\right)|$.

To determine the numerical FRFs, a modal analysis was performed by the finite-element software Ansys Mechanical APDL (Ansys, Inc., Canonsburg, PA, USA) and by modal decomposition the State-Space model of the modal system was obtained in Matlab (MathWorks) environment, deducing rapidly the frequency response functions knowing the strain ($\left[\Phi^{\epsilon }\right]\;\left(6\;\times \;m\right)$ ) and displacement mode shapes ([Φ](3×m)) for each element and node of the finite-element model, considering *m* modes (see [45,50]). The amplitude $|H_{\epsilon F}\left(f\right)|$ was determined averaging the longitudinal strain mode shapes for each mode on the sensor’s volume.

The longitudinal elastic modulus *E* of the 3D-printed specimens was obtained updating the FEM model (varying the elastic modulus itself) until the amplitude of the numerical frequency response function $H_{AF}\left(f\right)$ matched the experimental one. The density ρ of the 3D-printed structures was determined computing the ratio between the samples’ mass, measured by a digital scale (of centigram accuracy), and the specimens’ volume, computed knowing the samples’ dimensions.

The numerical model was implemented in light of the consideration that the strain-mode shapes, used to determine $H_{\epsilon F}\left(f\right)$, are different from the displacement mode shapes [45], used to compute $H_{AF}\left(f\right)$. Therefore, a comparison between the experimental $|H_{\epsilon F}\left(f\right)|$ and $|H_{AF}\left(f\right)|$ amplitudes would not be reasonable.

## 3. Results and Discussion

### 3.1. Quasi-Static Calibration

**Figure 5:**
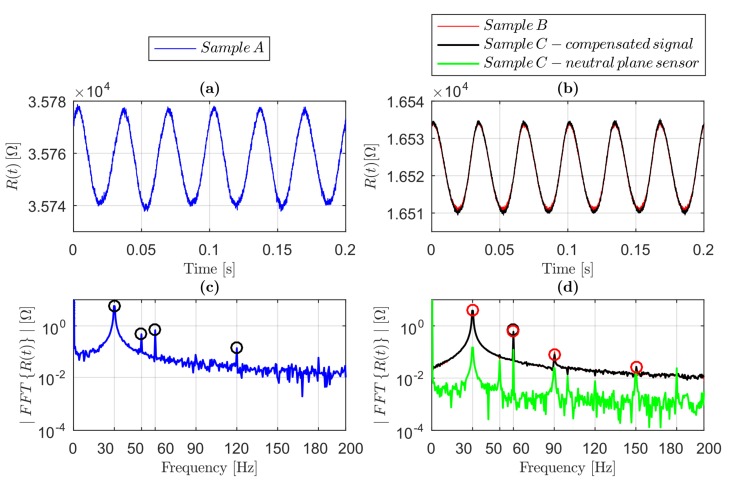
Sensor time responses R(t) and relative spectra under harmonic excitation of amplitude 4 N at the frequency of 30 Hz: (**a**) time response from Sample A; (**b**) time response from Samples B and C; (**c**) amplitude spectrum of the signal from Sample A; and (**d**) amplitude spectrum of the signals from Samples B and C (compensated and from the neutral plane).

Exciting the samples with harmonic loads at a frequency of 30 Hz, the response R(t) of the sensors is almost harmonic as well, as shown in Figure 5, in which the time responses and their corresponding amplitude spectra for Samples A (Figure 5a,c), B and C (Figure 5b,d) at a force amplitude of 4 N are shown.

From the amplitude spectrum of the signal from Sample A (Figure 5c), it was evident that the harmonic contribution at 30 Hz was greater than the amplitude of the signals from Samples B and C, even if the strain in Sample A was smaller. This is explainable if we consider that the sensor length in Sample A was 30 mm, while in Samples B and C it was 10 mm, hence $\bar{R}$ for Sample A is theoretically three times that of the one in Samples B and C. Therefore, the harmonic amplitude change in the resistance $R_{A}$ in Sample A was greater than in B and C. The higher harmonics of the fundamental frequency 30 Hz were in the sensor response, in particular in Sample A at 60 Hz and 120 Hz, while in Samples B and C, they were at 60 Hz, 90 Hz and 150 Hz, as highlighted in Figure 5c,d. The ratio between the magnitude of the spectrum (Figure 5c) at the frequency 60 Hz and at 30 Hz for Sample A was 12%, whereas for Samples B and C (Figure 5d) it was 17%, showing a higher level of non-linearity in the case of greater values of strain; this behavior was found for every amplitude of the input.

A source of noise due to EMI at 50 Hz and its integer multiples was found, as is clear in Figure 5c,d for Samples A and C (neutral plane signal), respectively. It is therefore reasonable to affirm that the EMI was due to the electrical grid. This noise in Sample A was found to be more relevant than in B and C, probably due to the greater sensor length of Sample A, resulting in an antenna-like functioning. In Figure 5d, there is evidence of the match between the amplitude spectrum of the signal in Samples B and C (compensated signal). Additionally, the neutral plane signal spectrum had peaks at the excitation frequency, at 50 Hz and their integer multiples. The spectrum amplitudes at 30 Hz, 50 Hz and their integer multiples were not negligible compared to the signals spectra of Samples B and C. This means that the sensor was not perfectly in the neutral plane, probably due to the printing resolution of 0.1 mm in height (one layer). The spectra evaluated at different force amplitudes ($F_{A}$) showed similar behavior.

**Figure 6:**
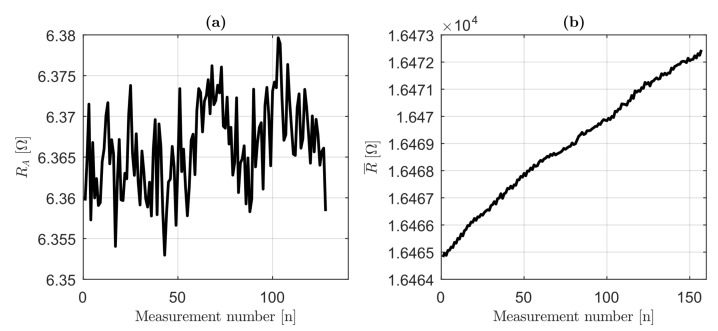
Long-term amplitudes and mean values of R(t) (harmonic load, frequency 30 Hz) for Sample B and force amplitude of 2.4 N: (**a**) $R_{A}$ collected values; and (**b**) $\bar{R}$ collected values.

Long-term (150 measurements of 1.6 s) $R_{A}$ and $\bar{R}$ measurements for Sample B at 2.4 N (30 Hz), at room temperature are shown in Figure 6a,b, respectively. The positive trend of $\bar{R}$ was clear but negligible (0.04%), while $R_{A}$ showed a slight (0.2%) deviation from the average value of 6.356. The assumptions of $R_{A}=const.$ and $\bar{R}=const.$ were reasonable; therefore, the temperature effects (i.e., internal heating due to damping or to Ohmic losses) were negligible.

**Figure 7:**
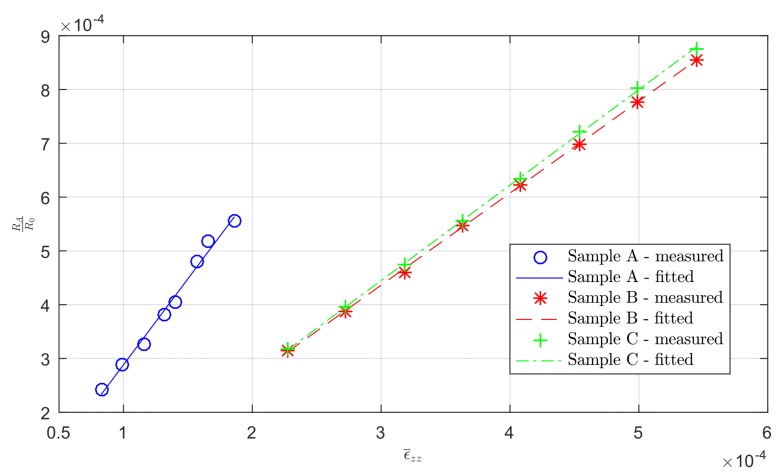
Quasi-static calibration curves estimating the strain using beam static theory for Samples A–C, excitation force amplitude of 2.4 N at frequency $f_{exc}=30$ Hz.

The validity of Equation (8) was tested for different excitation force amplitudes (as discussed before) and a clear linearity was confirmed (see Figure 7).

The coefficients of determination $R^{2}$ were 0.9870, 0.9998 and 0.9996 (for Samples A–C, respectively) for the Euler–Bernoulli beam theory estimated strain, showing a good estimation overall. The gauge factors (GFs) estimated for the sensors were 3.20, 1.71, and 1.77 for Samples A–C using the Euler–Bernoulli beam theory, respectively. The higher GF of sensor A is most likely due to the different geometry, i.e., the absence of greater width in the transversal parts (Figure 1) to reduce the cross sensitivity (other components of strain). The nonlinearities for the calibration curves were 6.49%, 1.02%, and 0.99% (Samples A–C, respectively) in the corresponding range of output measurements (Figure 7). The nonlinearity for the sensor in Sample A was much higher than for the others, probably be due to the transversal sensitivity. The values of $R_{0}$ used for Samples A–C were 35,758 Ω, 16,417 Ω and 16,453 Ω, respectively, measured as the mean values of the sensor responses at the beginning of the tests, assuming in this way the reference condition in the initial state of the sensor; the slight difference in $R_{0}$ between Samples B and C was due to the FDM 3D-printing uncertainty and to the different initial conditions of the tests.

### 3.2. Dynamic Measurements

**Figure 8:**
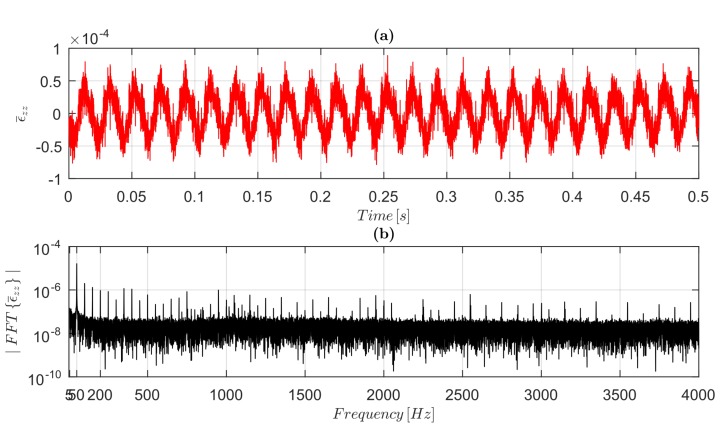
Broadband noise measurement in the range 5 Hz to 4000 kHz for the 3D-printed sensor in Sample B in the undeformed shape (without excitation force): (**a**) strain component ${\bar{\epsilon }}_{zz}\left(t\right)$ measured by the 3D-printed sensor; and (**b**) amplitude spectrum of ${\bar{\epsilon }}_{zz}\left(t\right)$.

The noise floor is fundamental to performing dynamic measurements; therefore, the broadband strain noise floor was determined for Samples B and C, which were used to prove the capability of the FDM 3D-co-printed strain sensors to perform dynamic measurements. Without applying loads, the rough signal of resistance R(t) (obtained by Equation (10) from E(t)) from the embedded sensors in Samples B and C was measured, and by using a gauge factor of 1.71 (the smallest value, obtained for Sample B), the strain noise ${\bar{\epsilon }}_{zz}\left(t\right)$ in the time domain (Figure 8a) and its relative amplitude spectrum (Figure 8b) were obtained.

The EMI’s contribution to the noise in the embedded sensors’ signals is also highlighted in Figure 8, where amplitude components at 50 Hz and its integer multiples are evident. A broadband strain noise floor of approximately 30 μϵ was determined.

**Figure 9:**
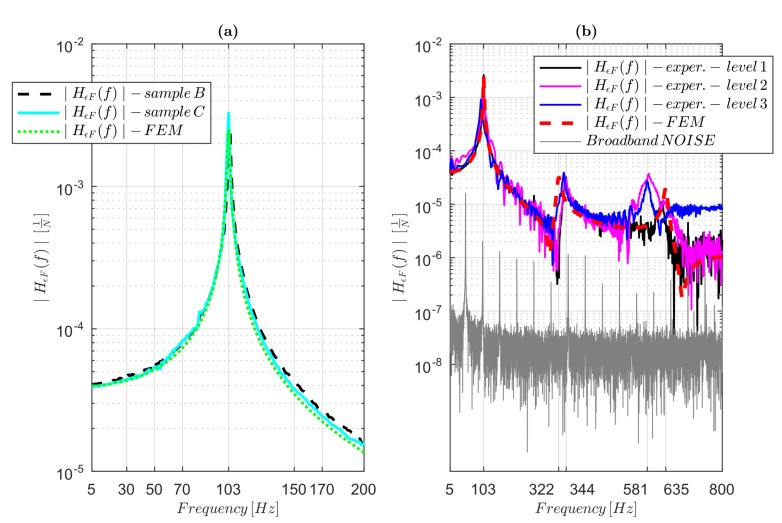
Dynamic embedded sensors’ validation, comparing experimental and numerical (FEM model) FRF amplitudes $|H_{\epsilon F}\left(f\right)|$: (**a**) experimental and numerical (FEM) strain FRF amplitudes $|H_{\epsilon F}\left(f\right)|$ comparing for Samples B and C in the range 5 Hz to 200 Hz; and (**b**) experimental and numerical strain FRFs (amplitudes) in the range 5 Hz to 800 Hz for Sample C (analogous results for Sample B).

To examine the possibility to perform dynamic strain measurements using FDM 3D-co-printed embedded sensors, the comparison between the FRFs’ amplitudes determined by the integrated sensors and the FRFs obtained using the numerical model was carried out, as shown in Figure 9. In particular, in Figure 9a, the experimental and numerical $|H_{\epsilon F}\left(f\right)|$ FRF amplitudes for Samples B and C are shown. The experimental FRF amplitudes for Samples B and C were computed using the *GFs* 3.37 and 3.50, respectively, obtained using the numerical model; the difference between these and the *GFs* computed theoretically (Euler–Bernoulli beam theory) is explainable by considering the boundary conditions, shear effects and the effective sensor shape taken into account in the FEM. In the range 5 Hz to 200 Hz, only the first natural frequency (103 Hz) for Samples B and C was present, as evident in Figure 9a. Additionally, the natural frequency was exactly matched when comparing the strain FRFs (amplitude). The sensors’ ability to measure dynamic strain in the range 5 Hz to 200 Hz for Samples B and C was therefore researched. The effects of the EMI noise-compensation method in Sample C were negligible, as evident in Figure 9a, comparing Samples B and C FRFs amplitudes; therefore, the same conclusions as for the quasi-static case can be obtained (showing only one result for Samples B and C in the next considerations). Exciting Samples B and C in the range 5 Hz to 4 kHz (using the sine sweep) at three different levels of force (from 1 to 3, the force root mean square increased), the experimental and numerical strain FRFs were estimated, as shown in Figure 9b.

The experimental $|H_{\epsilon F}\left(f\right)|$ amplitudes were obtained by using the GFs for Sample C. The 3D-printed sensor FRFs matched the first and second system’s natural frequency, as highlighted in Figure 9b, comparing the experimental and numerical (validated model by the reference sensor) FRFs. The third natural frequency can be considered matched: the difference between the FEM and the experimental FRFs was due only to the numerical validation (in the experimental $|H_{AF}|$, the third frequency was exactly coincident with the 3D-printed sensor frequency identification). After the third system frequency, a large deviation between the experimental and numerical sensor response functions was found. The experimental FRF was influenced by the input force level, showing probable nonlinearities at frequencies higher than about 600 Hz, undermining the sensor’s dynamic measurements’ reliability at high frequencies. Additionally, since the experimental $|H_{AF}|$ amplitudes were not found to be affected at high nonlinearities, the differences in amplitude in the experimental $|H_{\epsilon F}|$ can be ascribed to the nonlinearities of the sensors themselves as well as the low SNR at high frequencies (see Section 2.3.2).

Figure 9b also plots the broadband noise, which evidently limits the SNR, due principally to the EMI’s contribution.

## 4. Conclusions

The capability of FDM 3D-printed embedded sensors to perform dynamic strain measurements was proven up to 800 Hz by using a high-dynamic-range accelerometer and a numerical model, as a reference sensor and a reference model, respectively. The theoretical model, validated by experimental data, was used to demonstrate the feasibility of calibrating the integrated strain sensors using quasi-static tests, also taking into account the temperature effects, which were revealed to be negligible in their amplitude ($R_{A}$) variation. The hypothesis of a zero-order model for the sensors was confirmed up to 800 Hz by the comparison between experimental and numerical FRFs in terms of strain. The contribution of electromagnetic interference to the strain noise floor was researched. Although the compensation of electromagnetic noise performed by printing one sensor on the neutral plane of a beam was researched, it was not found to be effective. Additionally, negligible piezoresistive nonlinearities in the quasi-static and dynamic measurements for the 3D-printed sensors were found as long as the sensor is in the linear-response region of the structure.

This work paves the way for new applications of 3D-printed piezoresistive embedded sensors in which dynamic measurements are fundamental.

## Abbreviations

The following abbreviations are used in this manuscript:

PLA	Polylactic acid
S/M-WCNTs	Single/Multi-walled carbon nanotubes
CB	Carbon Black
FDM	Fused Deposition Modeling
EMI	Electromagnetic interference
FRF	Frequency-Response Function
SNR	Signal-to-noise ratio
